# Investigation of *Rhizopus oligosporus* Metabolites in Fermented Wheat Bran and Its Bio Function in Alleviating Colitis in Mice Model

**DOI:** 10.3390/metabo14070359

**Published:** 2024-06-26

**Authors:** Afifah Zahra Agista, Yu-Shan Chien, Takuya Koseki, Hazuki Nagaoka, Takuto Ohnuma, Yusuke Ohsaki, Chiu-Li Yeh, Suh-Ching Yang, Ardiansyah, Slamet Budijanto, Michio Komai, Hitoshi Shirakawa

**Affiliations:** 1 Laboratory of Nutrition, Graduate School of Agricultural Science, Tohoku University, Sendai 980-8572, Japan; 2 Faculty of Agriculture, Yamagata University, Tsuruoka 997-8555, Japan; 3 International Education and Research Center for Food Agricultural Immunology, Graduate School of Agricultural Science, Tohoku University, Sendai 980-8572, Japan; 4 School of Nutrition and Health Sciences, Taipei Medical University, Taipei 110301, Taiwan; 5 Department of Food Technology, Universitas Bakrie, Jakarta 12920, Indonesia; ardiansyah.michwan@bakrie.ac.id; 6 Faculty of Agricultural Engineering and Technology, IPB University, Bogor 16680, Indonesia

**Keywords:** adenosine, colitis, fermented wheat bran, *Rhizopus oligosporus*, short-chain fatty acids

## Abstract

Wheat bran (WB) is a low-value by-product of the wheat milling industry. Solid-state fermentation with *Rhizopus oligosporus* is performed to improve WB’s nutritional quality (RH). Twenty-five mice (11-week-old C57BL/6N male mice) were divided into three groups. The first group was fed a control diet (n = 8), the second group a 10% WB-supplemented diet (n = 8), and the last group had a 10% RH-supplemented diet (n = 9). The diet treatment was administered for 4 days before dextran sodium sulfate (DSS, 3% in drinking water) was administered for 9 days. RH supplementation prevented bodyweight loss and reduced the disease activity index in mice. An increase in the level of SCFAs in mouse intestines was detected post-RH supplementation, suggesting that SCFAs might have contributed to its anti-colitis effect. Metabolome analysis was conducted to explore other bioactive compounds in RH. *R. oligosporus* fermentation significantly increased the amounts of ergothioneine, arginine, branched-chain amino acids, and adenosine in wheat bran. All of these compounds are known to have antioxidant and anti-inflammatory capacities. These bioactive compounds might also have contributed to the RH’s ability to ameliorate DSS-induced colitis.

## 1. Introduction

The world’s wheat production in 2021–2022 was forecasted to be around 785.8 million tonnes [1]. After the de-husking process, the wheat grains that undergo the milling process can then be separated into a bran section (14–16% of the grain), a germ or embryo section (2–3%), and an endosperm section (81–84%) [2]. Wheat bran (WB) has been recorded to contain various antioxidant compounds such as phenolic acids, carotenoids, tocopherols, and alkylresorcinols. It is also known to be high in dietary fibers (44.6% of the wheat bran weight). WB also contains various nutritional compounds such as methionine and cysteine, B group vitamins, vitamin E, carotenoids, and various minerals [2,3]. However, WB is also known to have low nutritional value, namely due to its high level of phytic acid (3116–5839 mg/100 g dry weight), which can obstruct the absorption of minerals including calcium, iron, magnesium, and zinc. WB phenolic compounds also usually exist in forms bound to indigestible polysaccharides, limiting their bioavailability [2].

Fermentation is a method of food preservation that may also increase the nutritional value of its substrate. *R. oligosporus* is a fungus commonly used in producing tempe, a fermented bean food from Indonesia. Fermentation with *R. oligosporus* has been recorded to eliminate the phytic acid content in tempe made from soybean, chickpea, white bean, black bean, red lentil, green lentil, and broad bean [4,5]. *R. oligosporus* fermentation has also been observed to release phenolic compounds from their bonded form and is able to degrade lignin, cellulose, and hemicellulose [6,7]. While the product of WB fermentation with *R. oligosporus* has not been extensively investigated, *R. oligosporus* fermentation has the potency to increase the nutritional value of WB.

Inflammatory bowel disease (IBD) involves chronic and remitting inflammation that occurs in the gastrointestinal tract [8,9,10]. Patients with IBD experience not only pain and discomfort, but also an increased risk of developing colorectal cancer [9], negative impacts on their quality of life and work productivity, impairments to social and interpersonal interactions, and an increase in health care resource utilization [10]. Therefore, a new treatment that can prevent the onset of IBD is highly sought.

Polyphenols are one subset of the substances that have been suggested as capable of ameliorating inflammatory bowel disease. Polyphenols curb the onset of reactive oxygen species that accumulate in inflamed intestines [11]. Therefore, the ability of *R. oligosporus* to release phenolic compounds from their bonded form in WB might influence fermented WB’s colitis-preventing ability. On top of that, the risk of IBD [12] and the occurrence of IBD are also associated with dietary fiber intake [13]. *R. oligosporus* fermentation was found to be able to degrade lignin, cellulose, and hemicellulose, possibly altering WB’s dietary fiber content and, consequently, its capacity to mitigate the onset of IBD.

This study aimed to evaluate whether *R. oligosporus*-fermented WB (RH) supplementation is able to ameliorate dextran sodium sulfate (DSS)-induced intestinal inflammation in mice, an animal model of IBD. Furthermore, since the effect of *R. oligosporus* fermentation on WB has not been documented, we also observed the different metabolites that arise from this fermentation process, with a specific recognition of compounds that may control the risk of IBD.

## 2. Materials and Methods

### 2.1. Wheat Bran Fermentation

WB (400–450 g) was mixed with water (1:1) and steamed at 121 °C for 20 min. After the steamed mixture cooled down to 30 °C, 0.1% (*w*/*w*) tempe starter was inoculated. This tempe starter was a commercial starter containing *R. microspores var. oligosporus*, also known as *R. oligosporus.* Originally developed by The Indonesian Institute of Science in 2001, it has been produced and commercialized under the name of Raprima by PT. Aneka Fermentasi Industri, Bandung, Indonesia. This particular starter is currently one of the most commonly used starters in tempe production in Indonesia, especially in the Java area [14,15,16].

The inoculated mixture was then incubated in a shallow, rectangular vat vessel (solid-state fermentation) in an incubator at 30 °C for 44 h. To maintain an adequate temperature and access to oxygen, the mixture was stirred several times throughout the day. This fermented mixture was then mixed with 5 times the amount of water and saccharified at 65 °C for 16 h. The fermented WB that was intended to be used in the compound analysis was filtered, while the batch that was used in the animal feed was left as it was. Afterward, both mixtures were lyophilized for 48 h. The fermented WB (RH for *R. oligosporus*-fermented WB) produced was then stored at −30 °C. The non-fermented WB was prepared using the same process without the starter inoculation.

### 2.2. Animal Experiment

Twenty-five mice (11-week-old C57BL/6N male) were housed in a pathogen-free environment at 23 ± 3 °C, with a relative humidity of 55 ± 10% and a 12 h/12 h light/dark cycle. After four days of the acclimation period, the mice were divided into three groups. The first group (n = 8) was administered a control diet, the second group (n = 8) was administered a 10% WB-supplemented diet, and the third group (n = 9) was fed a 10% RH-supplemented diet. The diet formulations are displayed in Table 1.

The diet was administered for four days before the mice’s drinking water was swapped with water that had been mixed with 3% DSS. At the end of the fourth day, fecal samples from each mouse were collected. DSS was administered for 9 days before the mice were euthanized. During the experiment period, the mice were observed, and the severity of the effect of DSS was scored using the disease activity index. The scoring system used to evaluate the disease activity index (DAI) is shown in Table 2. The diet treatments were continued until the termination point of the experiment. After euthanasia, serum, spleen, and colon samples were collected. This experiment was approved by the Animal Research–Animal Care Committee of Tohoku University, and the corresponding ethical approval code is 2019Ag011-04.

### 2.3. High-Performance Liquid Chromatography (HPLC) Analysis for Short-Chain Fatty Acids (SCFAs)

A fecal short-chain fatty acid analysis was conducted as mentioned by Islam et al. [17]. In short, the fecal sample was homogenized in a 10 mM NaOH solution containing crotonic acids as an internal standard. The homogenized mixture was then centrifuged. The supernatants were then collected and extracted with chloroform to remove the fat-soluble compounds. The remaining supernatants were then diluted with NaH_2_PO_4_ pH 2.7 and used in the analysis. The HPLC analysis was performed under isocratic conditions with NaH_2_PO_4_ as a mobile phase with an Atlantis T3 column (4.6 mm × 50 mm, 5 μm, Waters, Milford, MA, USA) at 30 °C. The flow rate of the mobile phase was 0.5 mL/minute, and the runtime for each sample was 35 min, with a 100 μL injection volume. Short-chain fatty acids (SCFAs) were detected with a UV detector at 214 nm.

### 2.4. Quantitative Reverse Transcriptase Mediated Polymerase Chain Reaction (qRT-PCR)

The analysis was performed similarly to previously stated methods. Briefly, the middle sections of colon tissues were homogenized with ISOGEN (Nippon Gene Co., Ltd., Tokyo, Japan). The RNA from the homogenized samples was then isolated with chloroform. The crude RNA was then processed with the Magnosphere UltraPure mRNA purification kit (Takara Bio Inc., Shiga, Japan) according to the manufacturer’s instructions. The list of primers is provided in Table 3.

### 2.5. Dietary Fiber Analysis

The dietary fiber analyses of the WB- and RH-supplemented diets were conducted based on the AOAC 2011.25 method.

### 2.6. Capillary Electrophoresis-Mass Spectrometry (CE-MS)

CE-MS was conducted following a method previously described by Oikawa et al. [18]. Briefly, the samples with the addition of an internal standard were suspended in MeOH and centrifuged, and the supernatants were then dispensed for analysis.

### 2.7. Statistical Analysis

Data are presented as means ± SE. The analysis was performed using Sigma Plot 12.5 and MetaboAnalyst 6.0. The data were analyzed with a Student’s *t*-test, one-way ANOVA, or two-way repeated measurement ANOVA. The Tukey–Kramer test was applied as a post hoc test. Significant differences between groups are denoted in each figure.

## 3. Results

### 3.1. Dietary RH Supplementation Prevented DSS-Induced Colitis

**Figure 1:**
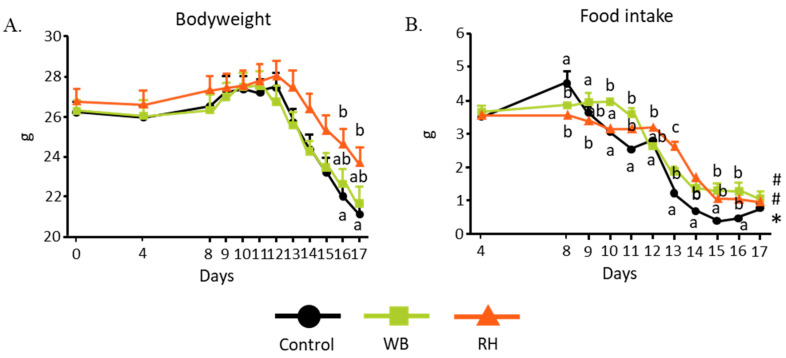
The effect of WB and RH on bodyweight and food intake. (**A**) RH supplementation protected mice from the bodyweight loss caused by DSS ingestion. WB- and RH-supplemented groups consumed more food compared to control group (**B**). Data were analyzed with two-way ANOVA (n = 8–9) and further analyzed with Tukey–Kramer post hoc test. Significant differences are denoted in each graph (*p* < 0.05, a, b, c, represent statistically different groups at the indicated *p*-value within the same day, and #, * represent statistically different groups at the indicated *p*-value within the overall experiment period).

**Figure 2:**
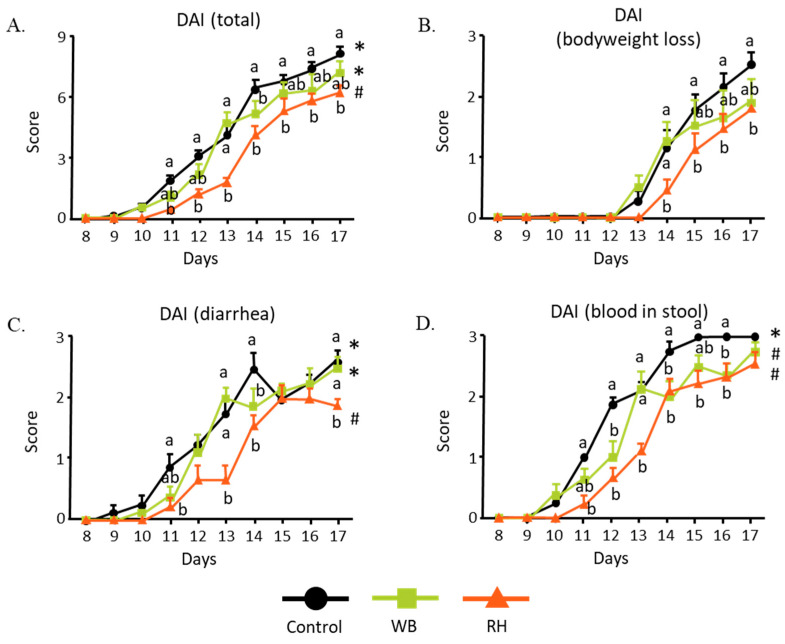
Compilation of several factors that comprise the disease activity index score (DAI). Total score of DAI (**A**). The DAI was scored based on the severity of bodyweight loss (**B**), diarrhea (**C**), and the presence of blood in stools (**D**). The data were analyzed with two-way ANOVA (n = 8–9) and further analyzed with a Tukey–Kramer post hoc test. Significant differences are denoted in each graph (*p* < 0.05, a, b, represent statistically different groups at the indicated *p*-value within the same day, and #, * represent statistically different groups at the indicated *p*-value within the overall experiment period).

DSS ingestion is known to cause inflammation in the intestine. Furthermore, the incorporation of DSS in the mice’s drinking water led to bodyweight reduction due to a combination of diarrhea, loss of blood, and loss of appetite. However, Figure 1A shows that the RH-supplemented diet group exhibited a significantly higher bodyweight in comparison to the mice that received the control diet. Both the RH- and WB-supplemented groups had significantly higher food intakes than the control group, as illustrated in Figure 1B. Figure 2A, however, displays a lower DAI value for the RH-supplemented group compared to those for the control group and WB-supplemented group. Since the DAI was scored based on bodyweight loss, the presence of blood in the stools, and diarrhea, we decided to take a closer look into each of these parameters. There were no differences in the bodyweight loss among all groups (Figure 2B). While both WB- and RH-supplemented diet intakes reduced the presence of blood in feces (Figure 2C), diarrhea was only ameliorated by RH supplementation (Figure 2D). This observation suggests the possibility that RH ingestion might be able to ameliorate the effect of DSS ingestion.

**Figure 3:**
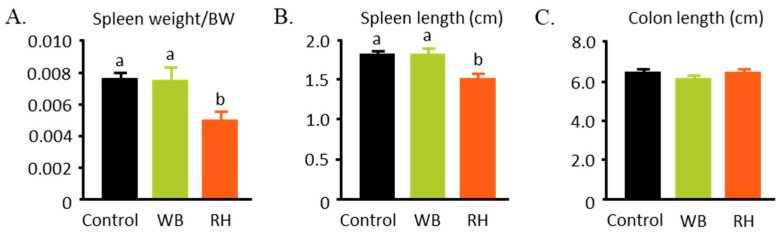
Organ comparison after DSS ingestion, followed by WB and RH supplementation. Spleen weight adjusted to bodyweight (**A**), spleen length (**B**), and colon length (**C**) was evaluated as a marker of the severity of DSS-induced colitis in mice. The data were analyzed with one-way ANOVA (n = 8–9) and further analyzed with a Tukey–Kramer post hoc test. Significant differences are denoted in each graph (*p* < 0.05, a, b represent statistically different groups at the indicated *p*-value).

DSS also commonly leads to spleen enlargement and colon shortening. The RH-supplemented group showed a significantly lower ratio of spleen weight to bodyweight and a shorter spleen length after DSS ingestion compared to the other groups (Figure 3A,B). However, no differences in the colon length were observed among the three groups (Figure 3C).

### 3.2. RH Supplementation Increases Mice Fecal SCFAs

**Figure 4:**
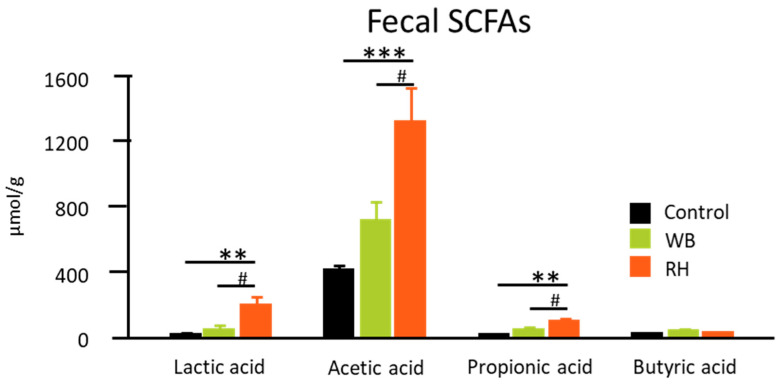
The SCFA content in fecal samples. The presence of lactic acid, acetic acid, propionic acid, and butyric acid in fresh fecal samples was analyzed. The data were analyzed with one-way ANOVA (n = 8–9) and further analyzed with a Tukey–Kramer post hoc test. Significant differences are denoted as ** (*p* < 0.01, comparison among all groups), *** (*p* < 0.001, comparison with control group), and # (*p* < 0.05, comparison with WB group).

WB is known to contain a high amount of dietary fiber (Table S1; data were calculated from the WB-supplemented diet, which consisted of 10% WB supplementation and 5% cellulose). This dietary fiber content can then be metabolized by either the gut microbiota or the fermenting microbes in fermented food to produce SCFAs. SCFAs are known to be able to play a key role in maintaining intestinal homeostasis. Furthermore, IBD patients have been reported to have lower amounts of SCFA-producing gut microbiota [19]. In this study, the fecal lactic acid, acetic acid, and propionic acid content levels were significantly higher in the RH-supplemented group than in the control or WB-supplemented groups (Figure 4). Fecal butyric acid content, however, showed no difference among all groups.

### 3.3. RH Consumption Affected the mRNA Levels of Il-17 and Il-22 in Colon

**Figure 5:**
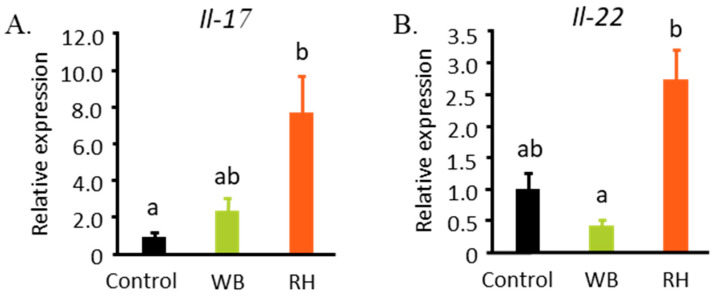
RH consumption possibly increased the mRNA expression of cytokines that maintain the intestinal barrier integrity. The mRNA levels of *Il-17* (**A**) and *Il-22* (**B**) were increased by RH supplementation. The data were analyzed with one-way ANOVA (n = 7–9) and further analyzed with a Tukey–Kramer post hoc test. Significant differences are denoted in each graph (*p* < 0.05, a, b represent statistically different groups at the indicated *p*-value).

**Figure 6:**
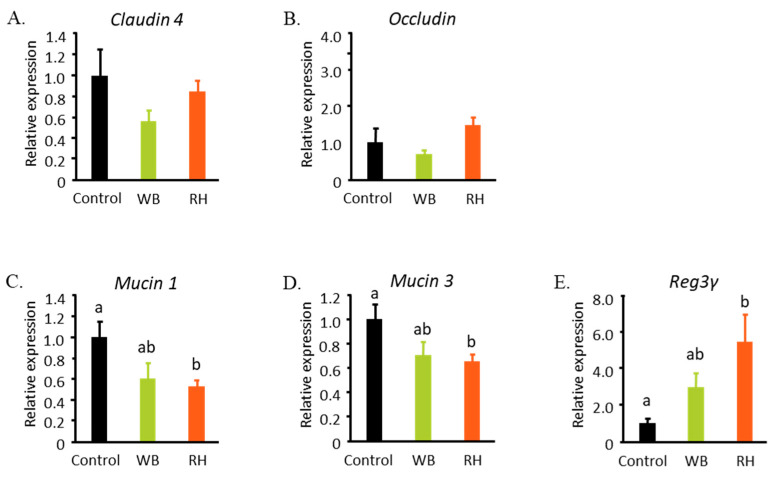
RH and WB supplementation affect the intestinal tight junction components and anti-bacterial peptides in the colon. The mRNA expressions of intestinal tight junction components such as *Claudin 4* (**A**) and *Occludin* (**B**) were not affected by the supplementations. However, the expressions of *Mucin 1* (**C**) and *Mucin 3* (**D**) were decreased by RH supplementation, while *Reg3γ* was increased (**E**). The data were analyzed with one-way ANOVA (n = 7–9) and further analyzed with a Tukey–Kramer post hoc test. Significant differences are denoted in each graph (*p* < 0.05, a, b represent statistically different groups at the indicated *p*-value).

SCFAs are known to be able to regulate the development and function of Th17 [20,21]. Here, we showed that the ingestion of an RH-supplemented diet increased the mRNA levels of *Il-17* and *Il-22*, as shown in Figure 5A,B. Both of these cytokines serve as key factors in maintaining the intestinal barrier and homeostasis [22]. While no differences were observed in the mRNA levels of intestinal tight junction components such as *Claudin 4* and *Occludin*, RH supplementation regulated the expression of anti-bacterial peptides in the large intestine, such as *Mucin 1*, *Mucin 3*, and *Reg3γ* (Figure 6).

### 3.4. Metabolomics Analysis of WB and RH

**Figure 7:**
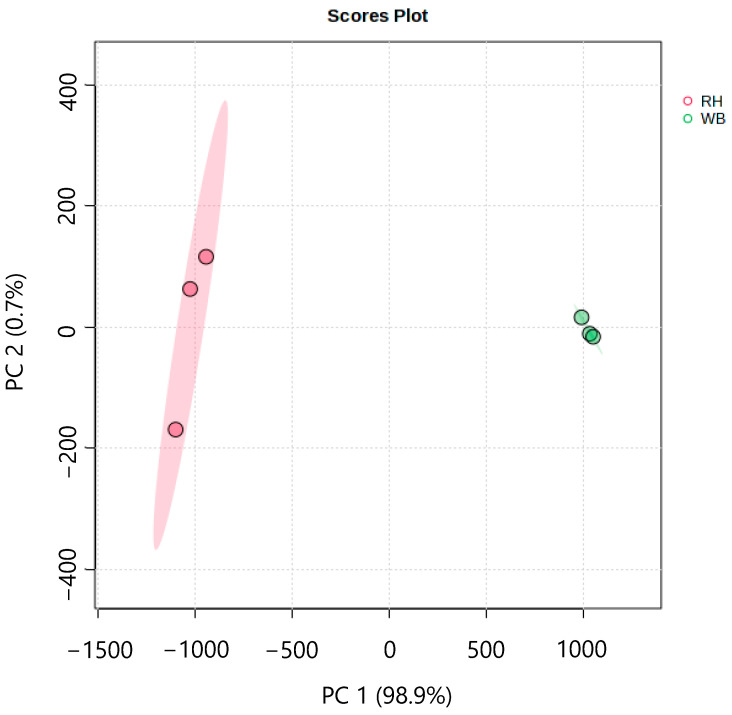
The effect of fermentation on wheat bran. RH: *R. oligosporus*-fermented wheat bran, and WB: non-fermented wheat bran.

To understand the effect of fermentation with *R. oligosporus* on the nutritional value of WB, a metabolomics analysis of *R. oligosporus*-fermented wheat bran was conducted. The principal component analysis (PCA) results of metabolites from non-fermented wheat bran (WB) and *R. oligosporus*-fermented WB (RH) are shown in Figure 7. The proportions of contributions of PC1 and PC2 are 98.9% and 0.7%, respectively. A score plot from this analysis shows that wheat bran fermented with *R. oligosporus* is different from non-fermented WB on PC1.

**Figure 8:**
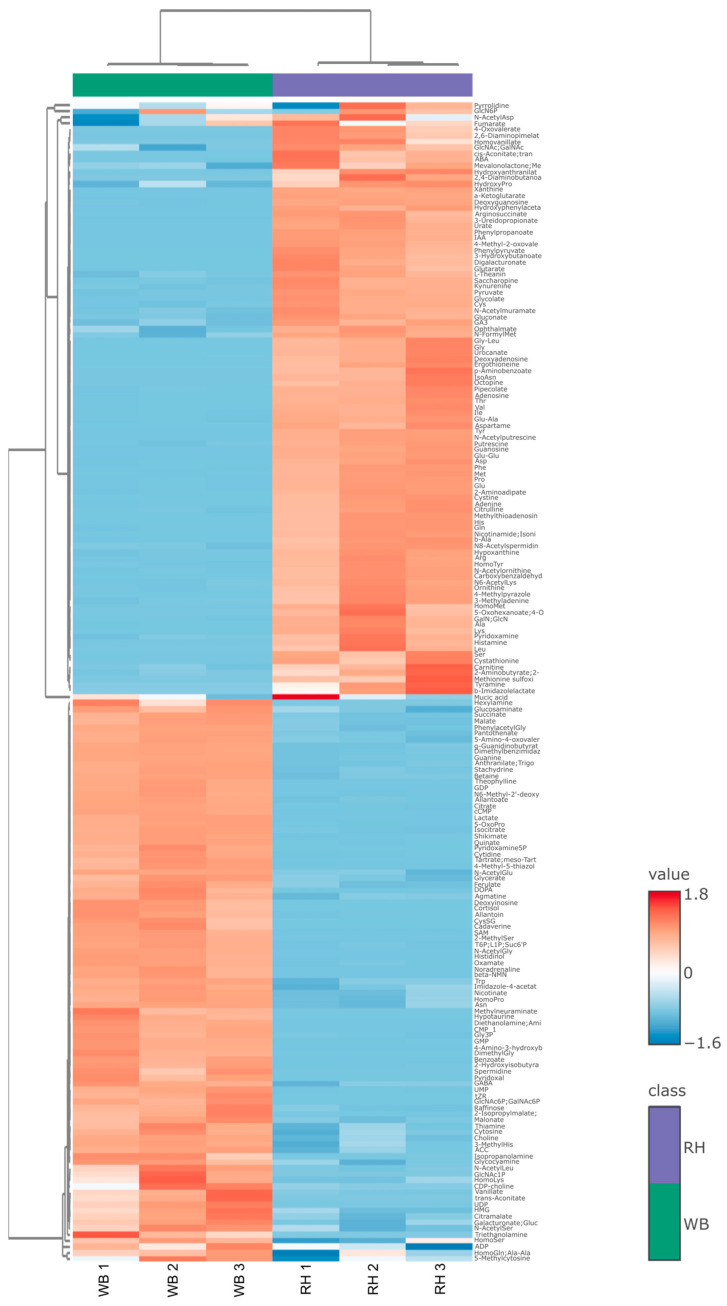
Comparison of the contents in WB and RH. Fermentation significantly changes the metabolite composition found in wheat bran.

This result is further elucidated in Figure 8, which shows the heatmap of the metabolite concentrations in WB and RH. It is apparent that fermentation changes some metabolites, such as the levels of various amino acids. *R. oligosporus* fermentation also decreased the levels of sugars such as raffinose, along with acids such as lactate, citrate, and succinate.

### 3.5. Bioactive Compounds in Fermented Wheat Bran

**Figure 9:**
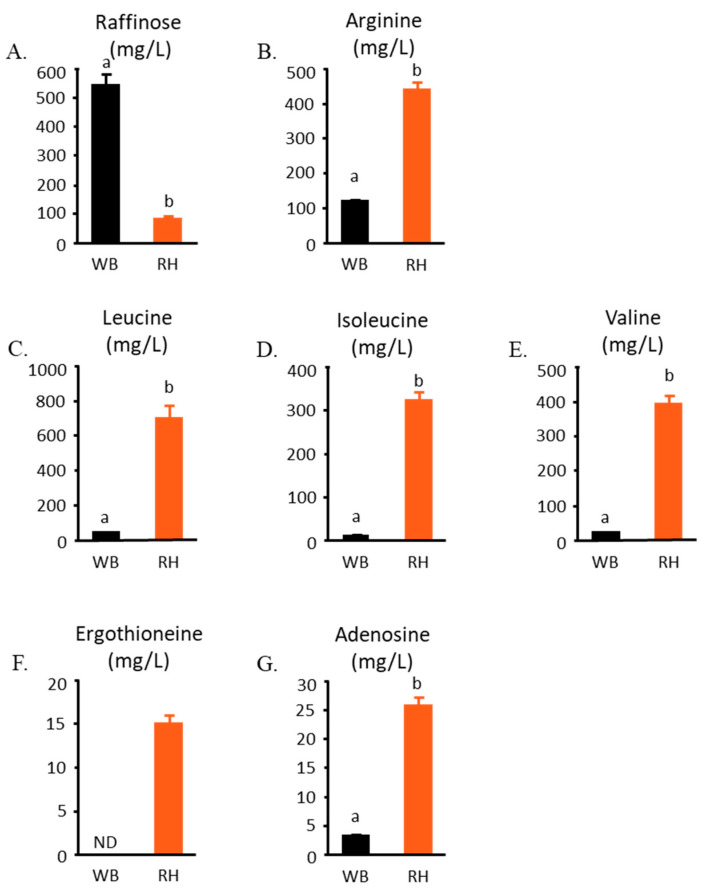
The effect of fermentation with *R. oligosporus* on wheat bran’s active compounds. (**A**) Raffinose, (**B**) arginine, (**C**) leucine, (**D**) isoleucine, (**E**) valine, (**F**) ergothioneine, and (**G**) adenosine are compounds that are known to be antioxidants and have anti-inflammatory properties. ND value signifies non-detectable compounds analyzed with the previously described method. The data were analyzed with *t*-tests. Significant differences are denoted in each graph (*p* < 0.05, a, b represent statistically different groups at the indicated *p*-value).

Whether *R. oligosporus*-fermented product has any health benefit has not been extensively explored. Figure 9 illustrates some of the bioactive compounds that can be found in WB and RH. One of the bioactive compounds that was identified in WB is raffinose. Raffinose was found to decrease due to *R. oligosporus* fermentation (Figure 9A). *R. oligosporus* fermentation also significantly produces compounds that are known to be able to exert anti-inflammatory effects or regulate inflammatory responses, namely arginine, isoleucine, leucine, valine, ergothioneine, and adenosine (Figure 9B–G). These bioactive compounds might have contributed to RH’s function in alleviating DSS-induced colitis.

## 4. Discussion

DSS-induced colitis in mice is a model that emulates the symptoms of IBD in humans. IBD involves chronic and remitting inflammation that occurs in the gastrointestinal tract [8,9,10]. The devastating impact that this disease has on the quality of life of its patients means that the treatment of IBD is highly sought [8,9,10]. In this paper, we illustrated that RH dietary supplementation is able to lower the effect of DSS in the large intestine. This effect appears to be related to RH’s ability to increase the level of *Il-22* in the colon. In the colon, IL-22, in turn, can induce the production of colonic REG3*γ* [22]. Here, we found that although the RH-supplemented diet failed to affect the intestinal tight junction markers, it elevated the mRNA levels of *Reg3γ.* At the same time, it also decreased the levels of *Mucin-1* and *Mucin-3*. The production of mucin is known to be induced by Il-17. We also observed an increase in *Il-17* mRNA expression due to RH supplementation. SCFAs are among the active compounds that are known to be able to affect the production of Il-22 and Il-17 in the intestine [19].

Wheat grain is known to have a high content of dietary fiber (9.2–17.0 g/100 g) [23]. Furthermore, *R. oligosporus* has been documented to be able to degrade various polysaccharides, and its fermentation has been described to increase the level of organic acids, such as acetic acid and propionic acid, in soybean [24]. During the fermentation process by *R. oligosporus*, dietary fiber in WB might be used by various microbiota to produce SCFAs. This suggestion is supported by the decrease in the low-molecular-weight dietary fiber content in the mouse diet supplemented by RH (Table S1). SCFAs can also be further produced in mouse intestines by the gut microbiota. SCFAs can normally act as a source of energy for intestinal epithelial cells, strengthen the intestinal barrier function, and regulate the intestinal immunity system. These organic acids are commonly known to signal through cell-surface G-protein coupled receptors (GPCRs) to modulate intestinal homeostasis [19]. However, the effect of RH supplementation on the expression of GPCR activity needs to be further studied.

While it is possible that the increases in *Il-17* and *Il-22* expressions after RH supplementation are induced by the SCFA content, other active compounds in RH might also play a role in maintaining the integrity of the intestinal membrane against DSS-induced colitis. The search for other bioactive compounds that are produced in RH was continued by employing a metabolome analysis approach. Figure 7 and Figure 8 elucidate the differences that arise between WB and RH due to *R. oligosporus* fermentation. *R. oligosporus* is capable of producing lipases, amylases, phytases, and galactosidase [7,25,26,27,28,29]. These enzyme functions can be seen in the depletion of simple sugars, such as raffinose and various acids, such as lactate, citrate, malonate, and shikimate, in WB. While raffinose is known to cause flatulence, it can also function as a prebiotic in human intestines [30]. *R. oligosporus* fermentation also produces various types of amino acids in abundance. The presence of various free amino acids in the fermented WB might be due to the ability of *R. oligosporus* to produce proteases and peptidases [7,26,27,28,29,30].

Among the abundance of amino acids that were produced by *R. oligosporus* in wheat bran is arginine. Arginine is a conditionally essential amino acid that functions as a precursor of nitric oxide in the human body. This amino acid protects against LPS-induced inflammation and even increases the content of glutathione peroxidase in LPS-stimulated cells, further decreasing the production of ROS and malonaldehyde [31,32]. Increases in other amino acids such as valine, leucine, and isoleucine were also detected in *R. oligosporus*-fermented wheat bran. These three amino acids belong to the branched-chain amino acid (BCAA) category and have been shown to reduce inflammatory responses. Isoleucine in particular is able to reduce the DSS-induced intestinal inflammation by regulating the NFκB pathway activation and maintaining the expression of intestinal tight junction components *Zo-1* and *Claudin 1* [33,34].

Additionally, *R. oligosporus* fermentation produces ergothioneine and adenosine in wheat bran. Ergothioneine is a fungus-derived antioxidant that has been classified among the GRAS compounds by the US Food and Drug Administration in 2018 [35]. Adenosine is well known for its ability to regulate the immune cell’s response to an inflammatory inducer [36,37,38,39,40,41,42]. Adenosine receptors can be found ubiquitously throughout various immune cells. Neutrophil, for example, has been reported to express four adenosine receptors. The A1 receptor plays a role in neutrophil chemotaxis, induces its adhesion, and increases phagocytosis and the generation of ROS. However, other adenosine receptors, such as A2A, A2B, and A3, have shown inhibitory effects on these functions [36,43]. In macrophages, A2A and A2B activation inhibit the release of proinflammatory cytokines. Increasing the expression of Il-10 by macrophages is also possible, possibly stimulating M1 to M2 phenotype changes [36,42]. Adenosine receptor A2A is also abundantly expressed in ILC3 and has been reported to be able to increase the production of Il-22 [44]. It was also reported to induce Il-17 secretion by Th17 cells [45]. These reports suggested that the adenosine content of RH, along with its other bioactive compounds, such as ergothioneine, arginine, BCAAs, and also SCFAs might contribute to its colitis-alleviating effect.

## 5. Conclusions

The fermentation of WB with *R. oligosporus* increased its nutritional value, such as by increasing its free amino acid content and increasing some antioxidants and anti-inflammatory compounds. These changes in RH nutritional values, in turn, might play some role in the capacity of RH to alleviate DSS-induced colitis in mice. On top of this, the ingestion of an RH-supplemented diet increased the level of some SCFAs, such as lactic acid, acetic acid, and propionic acid, in mice feces. The increased levels of SCFAs consequently contribute to maintaining the intestinal barrier and homeostasis by regulating the Il-17- and Il-22-producing cells’ activity.

## Figures and Tables

**Table 1:** Diet composition.

Ingredients	CON *	WB	RH
*tert*-Butylhydroquinone	0.008	0.0072	0.0072
Cystine	1.8	1.62	1.62
Choline bitartrate	2.5	2.25	2.25
Vitamin mixture	10	9	9
Mineral mixture	35	31.5	31.5
Soybean oil	40	36	36
Cellulose	50	45	45
Sucrose	100	90	90
Casein	140	126	126
α-Corn starch	155	139.5	139.5
Corn starch	465.70	419.12	419.12
WB	-	100	-
RH	-	-	100
Total	1000	1000	1000

* Control diet (CON), wheat bran supplemented diet (WB), and *R. oligosporus* supplemented diet (RH).

**Table 2:** Disease activity index score.

Score	Bodyweight Loss	Bloody Stool	Diarrhea
0	<5%	Normal	Normal
1	5–10%	Brown	Soft
2	10–20%	Reddish	Very soft
3	>20%	Bloody	Watery

**Table 3:** Primer list.

Genes		Sequences
Interleukin 17 (*Il-17*)	Forward	5′-CTC CAG AAG GCC CTC AGACTAC-3′
	Reverse	5′-GCT TTC CCT CCG CAT TGA CACAG-3′
Interleukin 22 (*Il-22*)	Forward	5′-GGAGACAGTGAAAAAGCTTG-3′
	Reverse	5′-AGCTTCTTCTCGCTCAGACG-3′
*Claudin-4*	Forward	5′-CCTCTGGATGAACTGCGTGGTG-3′
	Reverse	5′-GTCGCGGATGACGTTGTGAG-3′
*Occludin*	Forward	5′-CTTCTGCTTCATCGCTTCC-3′
	Reverse	5′-CTTGCCCTTTCCTGCTTTC-3′
*Mucin-1*	Forward	5′-TCGTCTATTTCCTTGCCCTG-3′
	Reverse	5′-ATTACCTGCCGAAACCTCCT-3′
*Mucin-3*	Forward	5′-CGTGGTCAACTGCGAGAATGG-3′
	Reverse	5′-CGTGGTCAACTGCGAGAATGG-3′
Regenerating islet-derived protein 3 γ (*Reg3γ*)	Forward	5′-TTCCTGTCCTCCATGATCAAAA-3′
	Reverse	5′-CATCCACCTCTGTTGGGTTCA-3′

## Data Availability

The datasets supporting the findings and the conclusions of this study are available within the article.

## Supplementary Materials

The following supporting information can be downloaded at https://www.mdpi.com/article/10.3390/metabo14070359/s1, Table S1: Dietary fiber composition of WB- and RH-supplemented diets.
