# Supplementary material for: Investigation of Rhizopus oligosporus Metabolites in Fermented Wheat Bran and Its Bio Function in Alleviating Colitis in Mice Model
Source: Metabolites. 2024 Jun 26;14(7):359. doi: 10.3390/metabo14070359 (PMC11278778; doi:10.3390/metabo14070359)
Supplement: Supplementary file 1 [file metabolites-14-00359-s001.zip › metabolites-3055762-supplementary.pdf]

Supplementary Table S1. Dietary fiber composition of WB and RH supplemented diet.

**Fiber composition**	**WB**	**RH**
Dietary fiber	12.6%	12.3%
Non water soluble dietary fiber	9.0%	9.0%
High molecular weight dietary fiber	2.2%	2.2%
Low molecular weight dietary fiber	1.4%	1.1%
